# Static and Dynamic Mechanical Behaviors of Electrostatic MEMS Resonator with Surface Processing Error

**DOI:** 10.3390/mi9010034

**Published:** 2018-01-17

**Authors:** Jingjing Feng, Cheng Liu, Wei Zhang, Shuying Hao

**Affiliations:** 1 Tianjin Key Laboratory for Advanced Mechatronic System Design and Intelligent Control, School of Mechanical Engineering, Tianjin University of Technology, Tianjin 300384, China; 163110303@stud.tjut.edu.cn; 2 Beijing Key Laboratory on Nonlinear Vibrations and Strength of Mechanical Structures, Beijing University of Technology, College of Mechanical Engineering, Beijing 100124, China; 3 National Demonstration Center for Experimental Mechanical and Electrical Engineering Education, Tianjin University of Technology, Tianjin 300384, China

**Keywords:** MEMS, processing error, section parameter, nonlinear vibration

## Abstract

The micro-electro-mechanical system (MEMS) resonator developed based on surface processing technology usually changes the section shape either due to excessive etching or insufficient etching. In this paper, a section parameter is proposed to describe the microbeam changes in the upper and lower sections. The effect of section change on the mechanical properties is studied analytically and verified through numerical and finite element solutions. A doubly-clamped microbeam-based resonator, which is actuated by an electrode on one side, is investigated. The higher-order model is derived without neglecting the effects of neutral plane stretching and electrostatic nonlinearity. Further, the Galerkin method and Newton–Cotes method are used to reduce the complexity and order of the derived model. First of all, the influence of microbeam shape and gap variation on the static pull-in are studied. Then, the dynamic analysis of the system is investigated. The method of multiple scales (MMS) is applied to determine the response of the system for small amplitude vibrations. The relationship between the microbeam shape and the frequency response is discussed. Results show that the change of section and gap distance can make the vibration soften, harden, and so on. Furthermore, when the amplitude of vibration is large, the frequency response softening effect is weakened by the MMS. If the nonlinearity shows hardening-type behavior at the beginning, with the increase of the amplitude, the frequency response will shift from hardening to softening behavior. The large amplitude in-well motions are studied to investigate the transitions between hardening and softening behaviors. Finally, the finite element analysis using COMSOL software (COMSOL Inc., Stockholm, Sweden) is carried out to verify the theoretical results, and the two results are very close to each other in the stable region.

## 1. Introduction

Electrostatically-actuated microbeams have become major components in many micro-electro-mechanical system (MEMS) devices [1] such as switches [2,3], sensors [4,5] and resonators [6] due to their geometric simplicity, broad applicability and easy to implement characteristics. Moreover, the existence of structure nonlinearity and nonlinear electrostatic force can make microbeams exhibit rich static and dynamic behaviors [7,8]. These behaviors have aroused the interest of many scholars, who have joined the study of MEMS. However, most of them have been aiming at the equal section beam under ideal conditions. However, the microbeams and microdiaphragms fabricated through surface processing technology are prone to errors during fabrication [9]. Such errors during fabrication of microdevices cannot be ignored as they can cause the bending of the microbeam neutral surface and change the width, thickness and gap distance of the microresonator. Hence, it is very important to analyze the static and dynamic behaviors of the electrostatically-actuated beam with surface processing error for understanding its global dynamic behavior, developing a dynamic control problem and optimizing vibration design. In the present paper, a doubly-clamped beam of variable thickness actuated by a one-sided electrode is considered to study the influence of section variation on static and dynamic behaviors.

The static pull-in instability is one of the key issues in the design of MEMS [10,11]. When the direct current (DC) voltage is increased beyond a critical value, the stable equilibrium positions of the microbeam cease to exist, and the pull-in instability will be triggered [12,13]. For example, Abdel-Rahman et al. [14] investigated an electrically-actuated microbeam accounting for midplane stretching and derived the static pull-in position of microbeam. Younis et al. [15] studied the effect of residual stress on the static pull-in of microresonators and found that the residual stress would increase the pull-in voltage. The system’s vibrations can cause an interesting nonlinear phenomenon such as hysteresis, softening behavior, snap through and dynamic pull-in instability when it is excited with DC and alternating current (AC) voltages [16,17,18]. These analyses are helpful to further grasp the dynamic instability of microcomponents. Ghayesh and Farokh [19] investigated the static and dynamic behavior of an electrically-actuated MEMS resonator based on the modified couple stress theory. It is found that the pull-in voltage is larger by the coupled correction theory compared with the classical theory. Zhang et al. [20] used the method of multiple scales (MMS) to study the response and dynamic behaviors of the resonant parameters resonant in the MEMS resonator. The softening behavior of the DC voltage and the effect of damping on the frequency response curve were discussed. Ibrahim [21] investigated the effect of nonlinearities of a capacitive accelerometer due to squeeze film damping (SQFD) and electrostatic actuation by the theoretical and experimental methods. Theoretical results are compared to experimental data showing excellent agreement. Ghayesh, Farokh and Gholipour [22,23] investigated the nonlinear dynamics of a microplate based on the modified couple stress theory. The influence of system parameters on the resonant responses was highlighted by the frequency-response and force-response curves. Alsaleem et al. [24] conducted an experimental study to understand the dynamic pull-in voltage of the electrostatic drive microresonator. The experimental and theoretical results are in good agreement. Furthermore, the applicable microresonator conditions are pointed out. However, the majority of the previous studies are based on the equal section microbeam, i.e., the impact of model errors is neglected.

With the deepening research in this discipline, the importance of understanding the processing error of the microbeam on its performance has been realized. There are several sources of errors possible, for example, residual [25], initial offset imperfection [26], surface processing technology precision [27], etc. The residual stress causes the bending of the microbeam to form a microarch [28]. Farokhi and Ghayesh [29,30] established the mathematical model of a geometrically imperfect microbeam/microplate, the nonlinear force of which was actuated on the basis of the modified couple stress theory. The influence of physical parameters on the natural frequency and frequency response were analyzed. What is more, Farokhi and Ghayesh [31] investigated the three-dimensional motion characteristics of perfect and imperfect Timoshenko microbeams under mechanical and thermal forces. Ruzziconi [32] studied many kinds of nonlinear behaviors of the microarch under different parameters through theoretical analysis. The conclusions are in good agreement with experimental analysis. Besides, a reliable theoretical model was obtained. Ruzziconi [33] predicted the global bifurcation of the electrostatically-actuated microarch and studied the complex dynamics of the microarch. Krylov et al. [34] studied the electrostatically-actuated microarch structures, focusing on the influence of system geometry parameters on its dynamic behaviors and found that the main influencing factors are microarch thickness, microarch height and the distance between the microarch and the plate. Xu et al. [35] studied the dynamic behavior of clamped-clamped carbon nanotubes with initial bending and explored the non-resonance and resonance of carbon nanotubes by the shooting method. Hassen et al. [36] established a clamped-clamped beam model considering the initial bending and obtained its static and dynamic response using the Galerkin method and MMS. The microbeam with initial offset imperfections is usually actuated by two electrodes. In this case, the microbeam is still rectangular. However, the initial offset imperfections can break the symmetry along the transverse vibrational direction in dynamic MEMS devices. Mobki et al. [37] discussed the influence of the initial offset imperfections on the static bifurcation of a MEMS resonator. Han et al. [38] considered the effect of initial offset imperfections on the mechanical behaviors of microbeam. The global static and dynamic analysis of the microresonator is carried out using MMS and the finite difference method. Results show that the initial offset may induce a complex frequency rebound phenomenon, and there exists the frequency response in the medium and large amplitude in-well transitions between softening and hardening behaviors. Although these two kinds of error forms have a great influence on the mechanical properties of the system, the microbeam model is still an equal section beam. The error caused due to the accuracy of surface machining will change the width or thickness of the microbeam. Such dimensional changes affect the structural stiffness and electrostatic force, so it is necessary to study these. However, the influence of such errors on the shape of the microbeam is random. Therefore, scholars usually do smoothing processing by setting up the parametric equation model and adjusting the shape of the microbeam by changing the parameters. Herrera [39] studied the resonant behavior of a single-layered variable section microbeam. Furthermore, scholars have attempted to optimize the MEMS device by optimizing the equation parameters. Joglekar and Trivedi [40,41] proposed a versatile parametric width function, which can smoothly vary the width of a clamped-clamped microbeam along its length. The parameters of the width function are optimized, and the methodology is demonstrated in several cases [40,41]. On this basis, Zhang [42] discussed the effects of the optimized shape on the dynamic response of the microbeam. Few researchers have considered the influence of variations in microbeam thickness on the mechanical behavior. Kuang and Chen [43] investigated the effect of shaping the thickness of a microactuator and gap distance on its natural frequencies. Their study concluded that the shape variation could significantly alter the dynamic behavior of the microbeam. In particular, the working voltage range was increased six times as compared to a uniform rectangular cross-section microbeam with a flat electrode. Najar et al. [27,44] simulated and analyzed the deflection and motion of variable section beams in MEMS devices, and the effect of changing their geometrical parameters on the static bifurcation and frequency response was observed. However, only single-sided section changes were considered in their study. The sections of the microbeam both change up and down by taking into account the actual processing result. One section change is merely applied to special cases. Therefore, in this paper, simultaneous changes in two sections of the microbeam are considered to ensure that the obtained research models will be closer to reality. In addition, only the static pull-in voltage and frequency response were studied in [27,44]. The nonlinear softening and hardening behaviors, spring softening and other non-linear behaviors are still unclear. It is also essential to study the scope of applications of the theoretical analysis. In this work, the influence of processing error on the nonlinear softening and hardening behaviors, electrostatic softening and dynamic behavior with a large amplitude are analyzed.

The structure of this paper is as follows. In Section 2, the model (partial differential equations) based on the electrostatically-driven microbeam considering the size effect is given. A parameter is proposed for describing the variations in the microbeam section to study the degree of influence. The Galerkin method and Newton–Cotes method are applied to transform the original equation into the ordinary differential equation. In Section 3, the effects of section parameters and gap distance on the static pull-in and safety area are discussed. In Section 4, the MMS is applied to determine the response of the system under small amplitude vibrations. The relationship between section parameters and nonlinear characteristics and the relationship between the section parameters and the transitions between softening and hardening are discussed. In Section 5, the results obtained using COMSOL (COMSOL Inc., Stockholm, Sweden) simulations are presented to verify the theory. Finally, the summary and conclusions are presented in the last section.

## 2. Mathematical Model

### 2.1. Governing Equation

In this paper, a model considering the effect of surface machining error on the thickness of the microbeam is studied. The bending vibration equation of the system is obtained through force analysis.

**Figure 1:**
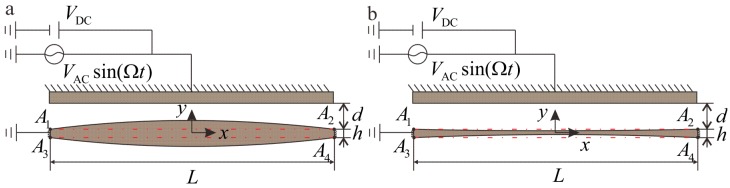
Schematic of an electrically-actuated microbeam. (**a**) *λ* > 0 case. (**b**) *λ* < 0 case. The dotted line in red is the ideal section location of the beam.

The schematic diagram of microbeam is shown in Figure 1. The thickness of the microbeam is not constant due to the processing errors. In this study, a section parameter *λ* is proposed. The shape of the microbeam is controlled by adjusting the value of section parameter *λ*. When *λ* < 0, the thickness of two clamped ends is greater than the thickness of the middle portion. When *λ* > 0, the thickness gradually decreases from the middle to both of the clamped ends. The *λ* = 0 case is the ideal case where the section beam thickness is uniform.

Since the pull-in behavior will cause structural damage, such instability should be avoided in microresonators. Stability can be ensured by considering the impact of processing errors on the pull-in effect.

The thickness of the microbeam changes according to $y_{1}(x)=\frac{h}{2}\;+\;\lambda h\sin\frac{\pi x}{L}$ and $y_{2}(x)=\;\left\{-\right\}(\frac{h}{2}\;+\;\lambda h\sin\frac{\pi x}{L})$ after considering the effect of surface processing error. *y*_1_(*x*) is a function consisting of the curve $\overset{⌢}{A_{1}A_{2}}$, and *y*_2_(*x*) is a function consisting of the curve $\overset{⌢}{A_{3}A_{4}}$. As shown in Figure 1, *A*_1_, *A*_2_ and *A*_3_, *A*_4_ represent the end points at the upper and lower sections, respectively. The parameter *λ* will be investigated to observe its impact on the system. The cross-sectional area $A(x)=A_{0}(1+2\lambda \sin(\frac{\pi x}{L}))$ and moment of inertia $I(x)=I_{0}{\left(1+2\lambda \sin(\frac{\pi x}{L})\right)}^{3}$ are calculated from *y*_1_(*x*) and *y*_2_(*x*). *A*_0_ = *bh* and $I_{0}=\frac{bh^{3}}{12}$ are the area and the moment of inertia of the two clamped sides, respectively. *L* and *b* are the length and the width of the microbeam, respectively. *h* is the thickness of the microbeam at the clamped ends. *c* is the damping coefficient of the system. *d* is the distance from the board to the *x*-axis. *E* is the effective Young’s modulus, and *ρ* is the material density. The actuation of the clamped-clamped microbeam is realized by using a bias voltage *V*_DC_ and AC voltage *V*_AC_ cos (Ω*t*). Ω is the alternating current excitation frequency. In the microresonator, *V*_AC_ is far less than *V*_DC_. *ε*_0_ is the dielectric constant in the free space, and *ε*_r_ is the relative permittivity of the gap space medium with respect to the free space.

The equation of motion that governs the transverse deflection *y*(*x,t*) is written as [27]:

(1) $$\frac{\partial^{2}}{\partial x^{2}}(EI(x)\frac{\partial^{2}y}{\partial x^{2}})+\rho A(x)\frac{\partial^{2}y}{\partial t^{2}}+c\frac{\partial y}{\partial t}=\frac{E}{2L}\int_{0}^{1}A(x){\left(\frac{\partial y}{\partial x}\right)}^{2}dx\frac{\partial^{2}y}{\partial x^{2}}+\frac{\varepsilon_{0}\varepsilon_{r}b{\left[V_{\mathrm{DC}}+V_{\mathrm{AC}}\cos(\Omega t)\right]}^{2}}{2{\left(d-y_{1}(x)-y\right)}^{2}}$$

The first term on the right-hand side of Equation (1) represents the mid-plane stretching effects, and the second term represents the electrostatic force. The following are the boundary conditions:

(2) $$y(0,t)=\frac{\partial y(0,t)}{\partial x}=0,\;y(L,t)=\frac{\partial y(L,t)}{\partial x}=0$$

The range of the parameter 6(*d*/*h*)^2^ is around 6(*d*/*h*)^2^∈ [0.1, 10] in the equal cross-section microbeam resonator [1]. It can be deduced from 6(*d*/*h*)^2^∈ [0.1, 10] that the ratio of the gap distance to the microbeam thickness ranges from 0.13–1.3 in equal cross-section microbeam resonator. After adding the parameter *λ*, the thickness and the gap distance become *h* + 2*λh* and *d* – *λh*, respectively. Therefore, the ratio becomes $0.13\leq \frac{d-\lambda h}{h+2\lambda h}\leq 1.3$. Besides, the range of *λ* should satisfy the physical model. When the upper and lower sections of the microbeam are becoming thinner, the change of section *λh* cannot exceed the microbeam’s neutral surface, which is $\lambda h>-\frac{h}{2}$. The change of section cannot contact the plate when the microbeam section is becoming thicker, which is $\lambda h<d-\frac{h}{2}$. Therefore, the ranges are as follows:

(3) $$\left\{ \begin{array}{l} 0.13\leq \frac{d-\lambda h}{h+2\lambda h}\leq 1.3\\ -\frac{h}{2}<\lambda h<d-\frac{h}{2} \end{array}\right.$$

It can be understood from Equation (3) that when *λ* = 0, the range of *d*/*h* is 0.12 ≤ *d*/*h* ≤ 1.3. By simplification:

(4) $$-0.3\leq 0.27\frac{d}{h}-0.37\leq \lambda \leq 0.79\frac{d}{h}-0.10\leq 0.9$$

For convenience, the following non-dimensional quantities are defined:

(5) $$\begin{array}{l} \overset{⌢}{x}=\frac{x}{L},\overset{⌢}{b}=\frac{b}{d},\overset{⌢}{y}=\frac{y}{d},{\overset{⌢}{y}}_{1}=\frac{y_{1}(x)}{d},{\overset{⌢}{y}}_{2}=\frac{y_{2}(x)}{d},\overset{⌢}{A}(\overset{⌢}{x})=\frac{A(x)}{A_{0}},\overset{⌢}{I}(\overset{⌢}{x})=\frac{I(x)}{I_{0}},\overset{⌢}{t}=\frac{t}{T},\omega =\frac{\Omega t}{\overset{⌢}{t}},\\ T=\sqrt{\frac{l^{4}\rho A_{0}}{EI_{0}}},\mu =\frac{cL^{4}}{EI_{0}T},\alpha_{1}=\frac{\varepsilon_{0}\varepsilon_{r}bl^{4}{V_{\mathrm{DC}}}^{2}}{2EI_{0}d^{3}},\rho =\frac{V_{\mathrm{AC}}}{V_{\mathrm{DC}}},\alpha_{2}=6{\left(\frac{d}{h}\right)}^{2} \end{array}$$

Substituting Equation (5) into Equations (1) and (2), the following non-dimensional equation of motion can be obtained:

(6) $$\frac{\partial^{2}}{\partial {\overset{⌢}{x}}^{2}}(I(\overset{⌢}{x})\frac{\partial^{2}\overset{⌢}{y}}{\partial {\overset{⌢}{x}}^{2}})+A(\overset{⌢}{x})\frac{\partial^{2}\overset{⌢}{y}}{\partial {\overset{⌢}{t}}^{2}}+\mu \frac{\partial \overset{⌢}{y}}{\partial \overset{⌢}{t}}-\alpha_{2}\int_{0}^{1}A(\overset{⌢}{x}){\left(\frac{\partial \overset{⌢}{y}}{\partial \overset{⌢}{x}}\right)}^{2}dx\frac{\partial^{2}\overset{⌢}{y}}{\partial {\overset{⌢}{x}}^{2}}=\frac{\alpha_{1}}{{\left(1-{\overset{⌢}{y}}_{1}(x)-\overset{⌢}{y}\right)}^{2}}$$

with boundary conditions:

(7) $$\overset{⌢}{y}(0,\overset{⌢}{t})=\frac{\partial \overset{⌢}{y}(0,\overset{⌢}{t})}{\partial \overset{⌢}{x}}=0,\;\overset{⌢}{y}(1,\overset{⌢}{t})=\frac{\partial \overset{⌢}{y}(1,\overset{⌢}{t})}{\partial \overset{⌢}{x}}=0$$

In the following simplifications, the “^” notation is dropped for convenience.

### 2.2. Galerkin Expansion

The Galerkin method is applied to derive a reduced-order model, and the deflection is expressed as:

(8) $$y(x,t)=\sum_{i=1}^{\infty }u_{i}(t)\phi_{i}(x)$$

The boundary conditions are as follows:

(9) $$\phi_{i}(0)=\phi_{i}(1)={\phi_{i}}^{\prime }(0)={\phi_{i}}^{\prime }(1)=0$$

where $u_{i}(t)$ is the modal coordinate amplitude of the *i*-th mode. $\phi_{i}(x)$ is the *i*-th mode shapes of the normalized undamped linear orthonormal. For an electrostatic actuated microbeam, a single degree-of-freedom model is sufficient to capture all the key nonlinear aspects in the Galerkin approximation [25]. However, the one-mode approximation cannot capture the mode coupling effect or internal resonances. These phenomena can be predicted to obtain a reasonable result by implementing the number of modes. Nevertheless, the analysis becomes computationally expensive. Since the main objective of this paper is to explore the main resonance problem in the nonlinear dynamics problem, the first-order mode is sufficient to obtain good results. In this paper, the first-order modal vibration y(x,t)=u(t)ϕ(x) is assumed. Substitute Equation (8) into Equation (6). Upon multiplying by $\phi_{i}(x)$ and integrating, the outcome is from *x* = 0 to 1, and one can obtain the following equation:

(10) $$\begin{array}{l} \ddot{u}+\mu \dot{u}+k_{1}u-\alpha_{2}k_{3}u^{3}=\alpha_{1}\int_{0}^{1}\frac{\phi (x)}{{\left(1-y_{1}(x)-\phi (x)u\right)}^{2}}dx+2\alpha_{1}\rho \cos(\omega t)\int_{0}^{1}\frac{\phi (x)}{{\left(1-y_{1}(x)-\phi (x)u\right)}^{2}}dx\\ \;+\alpha_{1}\rho^{2}\cos^{2}(\omega t)\int_{0}^{1}\frac{\phi (x)}{{\left(1-y_{1}(x)-\phi (x)u\right)}^{2}}dx \end{array}$$

where $\dot{u}=du/dt$. The symbolic meanings of *μ*, *k*_1_ and *k*_2_ are discussed in Appendix A.

### 2.3. Newton–Cotes Method

The integral of the electrostatic force in Equation (10) is complicated; therefore, the Newton–Cotes method is applied to fit the electrostatic force. 

The integral interval [$\tilde{a}$,$\tilde{b}$] is divided into n equal divisions. The step length is set as $\Delta h=\frac{\tilde{b}-\tilde{a}}{n}$. The node is $x_{k}=\tilde{a}+k\Delta h$, where *k* = 0, 1, 2, …, *n*. The interpolation type quadrature formula is as follows:

(11) $$\int_{\tilde{a}}^{\tilde{b}}\frac{\phi (x)}{{\left(1-y_{1}(x)-\phi (x)u\right)}^{2}}dx=(\tilde{b}-\tilde{a})\sum_{k=0}^{n}C_{k}^{\left(n\right)}\frac{\phi (x_{k})}{{\left(1-y_{1}(x_{k})-\phi (x_{k})u\right)}^{2}}$$

where $C_{k}^{n}$ is the Cotes coefficient, $C_{k}^{\left(n\right)}=\frac{1}{\tilde{b}-\tilde{a}}\int_{\tilde{a}}^{\tilde{b}}l_{k}(x)dx$, $l_{k}(x)=\int_{\tilde{a}}^{\tilde{b}}\prod_{j\neq k}\frac{\left(x-x_{j}\right)}{\left(x_{k}-x_{j}\right)}dx$.

Using the equipartition of nodes, the coordinates are transformed using $x=\tilde{a}+t\Delta h$. Using this transformation, the Cotes coefficients can be simplified further as:

(12) $$C_{k}^{\left(n\right)}=\frac{\Delta h}{\tilde{b}-\tilde{a}}\int_{0}^{n}\prod_{\begin{array}{l} j=0\\ j\neq k \end{array}}^{n}\frac{\left(t-j\right)}{\left(k-j\right)}dt=\frac{{\left(-1\right)}^{n-k}}{k!(n-k)!}\frac{1}{n}\int_{0}^{1}\prod_{\begin{array}{l} j=0\\ j\neq k \end{array}}^{n}\left(t-j\right)dt$$

**Figure 2:**
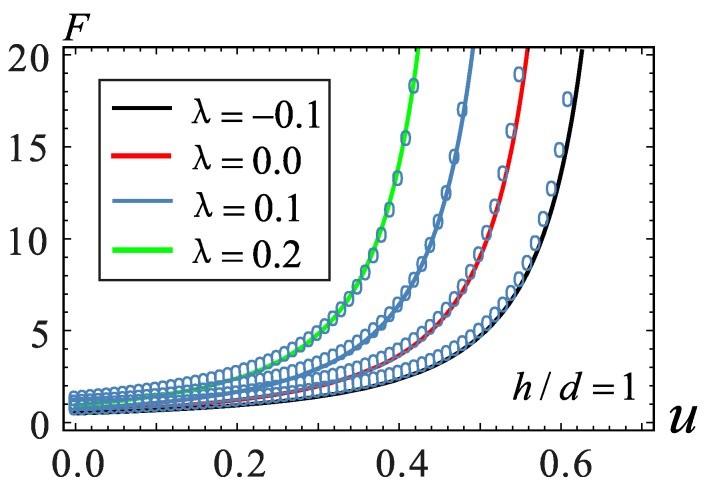
Contrast diagram of fitting curves under different section parameters. The circle is calculated using the numerical solution. The line is calculated using the Newton–Cotes method.

Through Equations (10)–(12), the simplified mathematical equation can be obtained.

(13) $$\begin{array}{l} \ddot{u}+\mu \dot{u}+k_{1}u-\alpha_{2}k_{3}u^{3}=\frac{0.61\alpha_{1}}{{\left(1-\delta \lambda -1.48u\right)}^{2}}+2\alpha_{1}\rho \cos(\omega t)\frac{0.61}{{\left(1-\delta \lambda -1.48u\right)}^{2}}\\ \;+\alpha_{1}\rho^{2}\cos^{2}(\omega t)\frac{0.61}{{\left(1-\delta \lambda -1.48u\right)}^{2}} \end{array}$$

where *δ* = *h*/*d*. It should be noted here that the maximum lateral displacement of the microbeam is at the midpoint viz., $y_{\max}=\phi (0.5)u\in [\lambda \delta ,1-\lambda \delta ]$. At the middle point of microbeam, the value of the modal function is ϕ(0.5)=1.59. Therefore, the range of *u* is $u\in [\frac{\lambda \delta }{1.59},\frac{1-\lambda \delta }{1.59}]$. The degree of matching is illustrated in Figure 2. The displacement is shown along the transverse coordinate and the electrostatic force along the ordinate.

## 3. Static Analysis

The equilibrium position and the maximum pull-in voltage of MEMS can be found through static analysis. The geometric parameters of the microbeam are *L* = 400 μm, *b* = 45 μm, *d* = 2 μm, *h* = 2 μm, *E* = 165 GPa, *ρ* = 2.33 × 10^3^ kg/m^3^ and the dielectric constant in free space *ε*_0_ = 8.85 × 10^−12^. Then, −0.1 ≤ *λ* ≤ 0.69 can be obtained from Equation (4). By removing the time-related items in Equation (13), the static response of the microresonator under the DC voltage actuation can be obtained.

(14) $$k_{1}u_{s}-\alpha_{2}k_{3}u_{s}^{3}=\frac{0.61\alpha_{1}}{{\left(1-\delta \lambda -1.48u_{s}\right)}^{2}}$$

**Figure 3:**
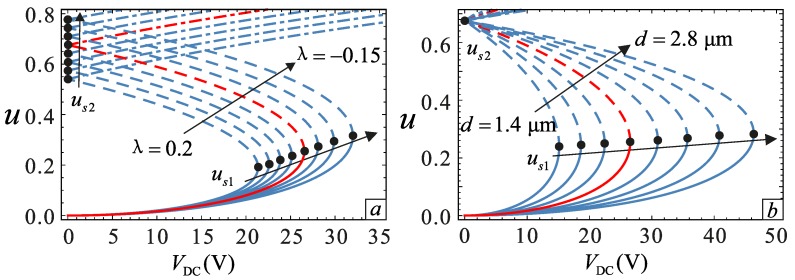
Relationship between the DC voltage and static equilibrium point under different physical parameters. (**a**) The influence of section parameters with *d* = 2.0 μm; (**b**) the influence of gap distance with *λ* = 0. The solid lines represent the stable solution. The dashed lines represent the unstable solution. The dotted lines are also stable, but it is impossible for them to appear in the physical model.

**Figure 4:**
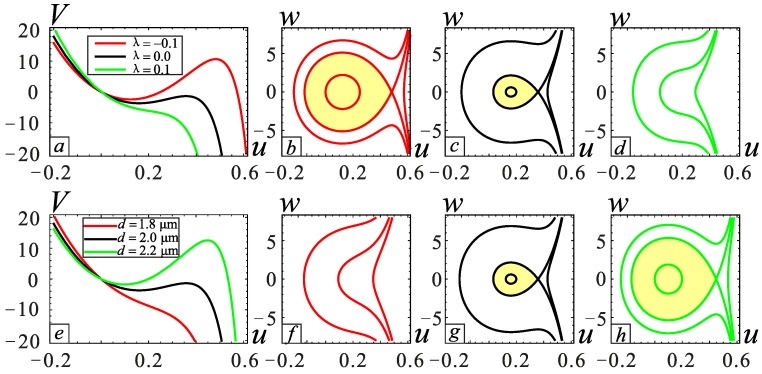
Potential energy curves and the corresponding phase diagrams under different physical parameters. (**a**) The potential energy curve under different section parameters with *d* = 2.0 μm; (**b**–**d**) are phase diagrams of *λ* = −0.1, *λ* = 0 and *λ* = 0.1, respectively; (**e**) the potential energy curve under different gap distances with *λ* = 0. (**f**–**h**) are the phase diagrams of *d* = 1.8 μm, *d* = 2.0 μm and *d* = 2.2 μm, respectively.

The relationship between the transverse displacement and DC voltage of the microbeam under different sections is shown in Figure 3a. With an increase of *λ*, the motion distance $u_{s2}$ of the microbeam gradually decreases. However, $u_{s2}$ has only a mathematical meaning and does not have any physical significance. In addition, the pull-in voltage and the pull-in location decreases as the *λ* increases. This is because the thickness of the beam increases as *λ* increases. As a result, the distance between the plate and the microbeam section decreases, which causes the axial movement distance to decrease resulting in pull-in. The influence of gap distance *d* on the static bifurcation is shown in Figure 3b. It can be seen from this figure that the equilibrium point $u_{s2}$ is almost unchanged. The pull-in location increases slowly with the increase of *d*, and this effect is opposite that of *λ*. Therefore, the study of the relationship between *λ* and *d* is very necessary. The operating voltage range of the microresonator can be predicted through analysis. When *V*_DC_ = 25 V, several cases are selected to observe in Figure 4, and the yellow regions are the stable regions. It can be found that *λ* promotes the pull-in phenomenon, whereas *d* inhibits the pull-in occurrence. The results are consistent with the situation in Figure 3.

## 4. Dynamic Analysis

The resonance frequency and bifurcation behavior can be obtained through dynamic analysis. The MMS is used to investigate the response of the microresonator with small vibration amplitude around the stable equilibrium positions. Introducing $u=u_{s}+u_{A}$, $u_{s}$ is the response to DC voltage and $u_{A}$ is the response to AC voltage. The response of DC voltage $u_{s}$ is obtained from Equation (14).

Substitute $u=u_{s}+u_{A}$ into Equation (13), and expand the electrostatic force equation up to third-order via Taylor expansion; the terms representing the equilibrium position can be eliminated. Since *V*_AC_ is far less than *V*_DC_ in the microresonator, the terms *V*_DC_ = *O*(1) and *V*_AC_ = *O*(*ε*^3^) are considered. Here, *ε* is regarded as a small non-dimensional bookkeeping parameter. Therefore, Equation (13) can be modified as:

(15) $${\ddot{u}}_{A}+\varepsilon^{2}\mu {\dot{u}}_{A}+\omega_{n}^{2}u_{A}+a_{q}{u_{A}}^{2}+a_{c}{u_{A}}^{3}=\varepsilon^{3}f\cos(\omega t)$$

The symbolic meanings of $\omega_{n}$ $a_{q}$ $a_{c}$ and f are presented in Appendix A.

(16) $$\omega =\omega_{n}+\varepsilon^{2}\sigma$$

The approximate solution of Equation (15) can be obtained in the following form:

(17) $$u_{A}(t,\varepsilon )=\varepsilon u_{A1}(T_{0},T_{1},T_{2})+\varepsilon^{2}u_{A2}(T_{0},T_{1},T_{2})+\varepsilon^{3}u_{A3}(T_{0},T_{1},T_{2})$$

where $T_{n}=\varepsilon^{n}t,$ *n* = 0, 1, 2.

Substituting Equations (16) and (17) into Equation (15) and equating the coefficients of like powers of *ε*, the following equations can be obtained:

(18) $$O(\varepsilon^{1}):D_{0}^{2}u_{A1}+\omega_{n}^{2}u_{A1}=0$$

(19) $$O(\varepsilon^{2}):D_{0}^{2}u_{A2}+\omega_{n}^{2}u_{A2}=-2D_{0}D_{1}u_{A1}-a_{q}u_{A1}^{2}$$

(20) $$\begin{array}{ll} O(\varepsilon^{3}): & D_{0}^{2}u_{A3}+\omega_{n}^{2}u_{A3}=-2D_{0}D_{1}u_{A2}-2D_{0}D_{2}u_{A1}-D_{1}^{2}u_{A1}\\ & -\mu D_{0}u_{A1}-2a_{q}u_{A1}u_{A2}-a_{c}u_{A1}^{3}\\ & +f\cos(\omega_{n}T_{0}+\sigma T_{2}) \end{array}$$

where $D_{n}=\frac{\partial }{\partial T_{n}}$, *n* = 0, 1, 2.

The general solution of Equation (18) can be written as:

(21) $$u_{A1}(T_{0},T_{1},T_{2})=A(T_{1},T_{2})e^{i\omega_{n}T_{0}}+\bar{A}(T_{1},T_{2})e^{-i\omega_{n}T_{0}}$$

Substituting Equation (21) into Equation (19), yields:

(22) $$D_{0}^{2}u_{A2}+\omega_{n}^{2}u_{A2}=-2i\omega_{n}\frac{\partial A}{\partial T_{1}}e^{i\omega_{n}T_{0}}-a_{q}(A^{2}e^{2i\omega_{n}T_{0}}+A\bar{A})+cc$$

where *cc* represents the complex conjugate terms.

To eliminate the secular term, one needs:

(23) $$-2i\omega_{n}\frac{\partial A}{\partial T_{1}}e^{i\omega_{n}T_{0}}=0$$

which indicates that *A* is only a function of *T*_2_.

Thus, Equation (22) becomes:

(24) $$D_{0}^{2}u_{A2}+\omega_{n}^{2}u_{A2}=-a_{q}(A^{2}e^{2i\omega_{n}T_{0}}+A\bar{A})+cc$$

The solution of $u_{A2}$ can be given as:

(25) $$u_{A2}(T_{0},T_{2})=\frac{a_{q}A^{2}}{3\omega_{n}^{2}}e^{2i\omega_{n}T_{0}}-\frac{a_{q}A\bar{A}}{\omega_{n}^{2}}+cc$$

Substituting Equations (21) and (25) into Equation (20) yields the secular terms:

(26) $$2i\omega_{n}\frac{\partial A}{\partial T_{1}}+\mu i\omega_{n}A-\frac{10a_{q}^{2}A^{2}\bar{A}}{3{\omega_{n}}^{2}}+3a_{c}A^{2}\bar{A}-\frac{f}{2}e^{i\sigma T_{2}}=0$$

At this point, it is convenient to express *A* in the polar form:

(27) $$A=\frac{1}{2}a(T_{2})e^{i\beta (T_{2})}+cc$$

Substituting Equation (27) into Equation (26) and separating the imaginary and real parts yield:

(28) $$\frac{Da}{DT_{2}}=-\frac{\mu }{2}a+\frac{f}{2\omega_{n}}\sin\varphi$$

(29) $$a\frac{D\varphi }{DT_{2}}=\sigma a+a^{3}(\frac{5a_{q}^{2}}{12\omega_{n}^{3}}-\frac{3a_{c}}{8\omega_{n}})+\frac{f}{2\omega_{n}}\cos\varphi$$

where $\varphi =\sigma T_{2}-\beta$.

The steady-state response can be obtained by imposing the conditions: $\frac{Da}{DT_{2}}=\frac{D\varphi }{DT_{2}}=0$. Finally, the frequency response equation can be derived as follows:

(30) $$a^{2}({\left(\frac{\mu }{2}\right)}^{2}+{\left(\sigma +a^{2}\kappa \right)}^{2})={\left(\frac{f}{2\omega_{n}}\right)}^{2}$$

where $\kappa =\frac{5a_{q}^{2}}{12\omega_{n}^{3}}-\frac{3a_{c}}{8\omega_{n}}$.

The vibration peak value and backbone curve can be decided by $a_{\max}=f/(\mu \omega_{n})$ and $\omega =\omega_{n}-\kappa a_{\max}$, respectively. The stability of the periodic solution can be determined by evaluating the eigenvalues of Jacobian matrix of Equations (28) and (29) at $\left(a_{0},\varphi_{0}\right)$.

(31) $$J=\left|\begin{array}{cc} -\frac{\mu }{2} & \frac{f}{2\omega_{n}}\cos\varphi_{0}\\ 2\kappa a_{0}-\frac{f}{2\omega_{n}a_{0}^{2}}\cos\varphi_{0} & -\frac{f}{2\omega_{n}a_{0}}\sin\varphi_{0} \end{array}\right|$$

The system is stable if all the eigenvalues are negative; otherwise, the system is unstable [45].

The important dynamic properties of the microresonators include resonant frequency, frequency response, pull-in behavior, and so on. These properties show a very significant influence on the performance of microresonators. Therefore, the MMS and numerical analysis are used to observe the dynamic behaviors of microresonator under the influence of various factors such as different geometric parameters, external excitation, and so on.

### 4.1. Dynamic Analysis with Small Amplitude

The soft and hard nonlinearities of the system are related to *ĸ*. Positive *ĸ* can lead to a softening-type behavior, while the negative value can lead to a hardening-type behavior. Meanwhile, when the *ĸ* value is approximately zero, i.e., the amplitude is small enough, the system experiences monostable vibration, i.e., linear-like vibration, which is an ideal state for MEMS designers. From Equation (30), one can notice that the nonlinear behaviors are affected by geometrical shape and electrostatic forces. The shape of the section is changed by adjusting the value of *λ*. Further, the relationship between the physical parameters and section shape is explored. The following physical quantities are assumed: *L* = 400 μm, *b* = 45 μm, *ρ* = 2.33 × 10^3^ kg/m^3^, *E* = 165 GPa, dielectric constant *ε*_0_ = 8.85 × 10^−12^ F/m and the clamped end thickness *h* = 2 μm. The other variation parameters are listed in Table 1. Next, the relationship between frequency response and various physical parameters is investigated.

**Figure 5:**
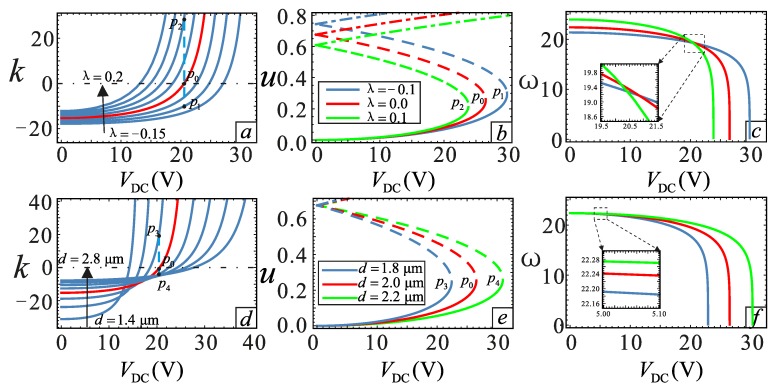
Relationship between DC voltage and mechanical behaviors under different physical parameters. (**a**–**c**) are the relationship between DC voltage and nondimensional parameter *ĸ*, static equilibrium and equivalent natural frequency, respectively, under different section parameters with *d* = 2.0 μm. (**d**–**f**) are the relationship between DC voltage and nondimensional parameter *ĸ*, static equilibrium and equivalent natural frequency, respectively, under different gap distance with *λ* = 0. The solid lines represent the stable solution. The dashed lines represent the unstable solution. The dotted lines also represent a stable case, but it is impossible for them to appear in the physical model.

Section changes due to the processing errors will have an impact on the system vibration. The effect of *λ* and *d* on the nonlinear behavior is shown in Figure 5, and the dimensionless damping coefficient *μ* = 0.1. *V*_DC_ = 20.5544 V is obtained by calculating *λ* = 0 and *ĸ* = 0. It can be noticed in Figure 5a,d that both *λ* and *d* can change the nonlinear soft and hard behavior. When *λ* = −0.1, the system has hard nonlinear characteristics. With the increase of *λ*, the system transits from hard to soft nonlinearity. *λ* = 0 is the dividing line. The parameters *λ* and *d* show an opposite effect. The points *P*_0_, *P*_1_, *P*_2_, *P*_3_ and *P*_4_ are selected for analysis. Figure 5c,f shows the relationship between DC voltage and equivalent frequency at different *λ* and *d*. It can be seen that at *V*_DC_ = 0, the larger the value of *λ* is, the higher is the equivalent natural frequency. This is because of the increase of system stiffness, which is caused by the increase of *λ*. One can see from Figure 5c,f that the pull-in phenomenon could be promoted with an increase in the value of *λ* and a decrease in the value of *d*. At the same time, with the increase of the DC voltage, the equivalent frequency decreases. There is a significant “spring softening” phenomenon. The greater the value of *λ* is, the more obvious the phenomenon becomes.

**Figure 6:**
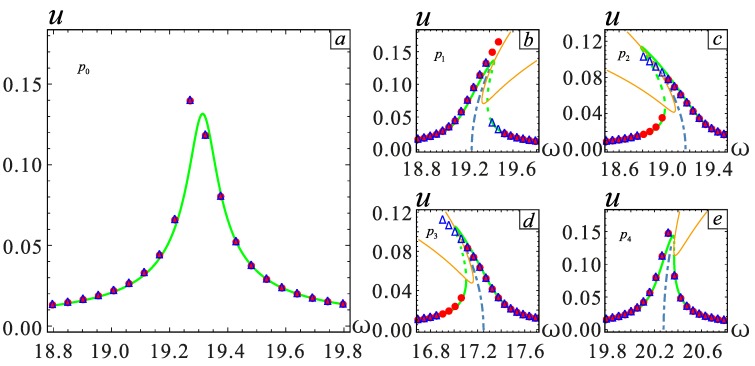
Frequency response curve in different situations, i.e., corresponding to cases *P*_0_, *P*_1_, *P*_2_, *P*_3_ and *P*_4_. (**a**) is the case of *P*_0_. (**b**) is the case of *P*_1_. (**c**) is the case of *P*_2_. (**d**) is the case of *P*_3_. (**e**) is the case of *P*_4_. The solid lines represent stable solutions. The dashed lines represent unstable solutions. The point represents the numerical solution.

To validate the above theoretical results, the frequency responses of five cases shown in Table 1 are studied using MMS. The long-time integration method of Equation (13) is used to obtain the numerical solutions. The accuracy of the results is verified by comparing both results. The AC excitation amplitude *V*_AC_ is varied to adjust the maximum amplitude. It can be known from Figure 6a–c that the nonlinear behavior changes from hardening to softening when *λ* = −0.1, *λ* = 0 and *λ* = 0.1. The system shows a hard nonlinearity behavior at *λ* = −0.1. When *λ* = 0.1, the system shows a soft nonlinearity behavior, and *λ* = 0 is the dividing line, where the vibration is linear.

In addition to *λ*, the gap distance *d* will also affect the frequency response. The parameters *λ* and *d* affect the soft and hard behavior in the opposite way. The nonlinear behavior changes from soft nonlinear to hard nonlinear when *d* = 1.8 μm, *d* = 2.0 μm and *d* = 2.2 μm, as shown in Figure 6a,d,e. Therefore, adjusting the relationship between *d* and *λ* to achieve linear behavior is necessary.

However, this time, the numerical and analytical solutions do not match at point *P*_0_. The numerical solution shows a softening behavior, whereas the analytical solution shows a linear behavior. This situation will be elaborated in detail below.

### 4.2. Dynamic Analysis with Large Amplitude

**Figure 7:**
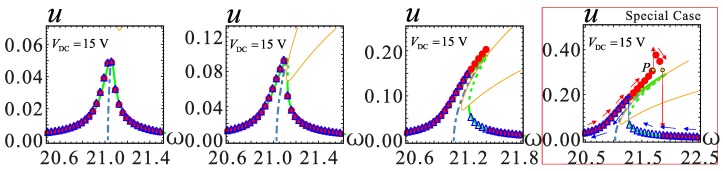

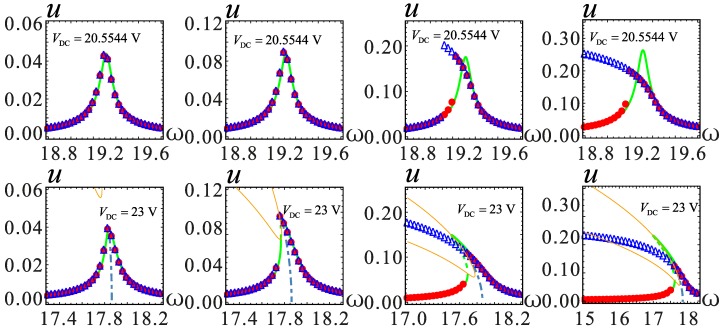
The frequency response changes with AC voltage at *λ* = 0. The DC voltages are *V*_DC_ = 15 V, *V*_DC_ = 20.5544 V and *V*_DC_ = 23 V, respectively, from top to bottom in the figure. The solid lines represent stable solutions. The dashed lines represent unstable solutions. The point represents a numerical solution.

With an increase of AC voltage, the MEMS resonator may undergo large amplitude vibration. When the AC voltage is increased to beyond a certain value, the analytical and numerical solutions will not match, for example, corresponding to the point *P*_0_ in Figure 6a. This phenomenon will be analyzed in detail in Figure 7. In addition, the *V*_DC_ = 15 V and *V*_DC_ = 23 V cases are considered to observe the phenomenon in the soft and hard nonlinearity cases. The amplitude of vibration increases, i.e., shifts from left to right, when the AC voltage is adjusted. When the vibration amplitude is small, the numerical and analytical solutions match very well. When the amplitude is close to *u* = 0.2, this difference between the solutions begins to appear. This phenomenon shows that the softening effect of analytic solutions is weakened. This is because the higher order terms in the Taylor expansion of the electrostatic force equation are omitted during the simplification process. These higher order terms are negligible when the amplitude is small. However, as the amplitude increases, these terms are not negligible; especially the frequency response in the red frame, which transits from hardening to softening behavior. When the vibration amplitude reaches around *u* = 0.3 and as the DC voltage increases, the influence of electrostatic force nonlinearity exceeds the structural stiffness nonlinearity. At this time, the electrostatic force plays a leading role. During sweep frequency response analysis, the resonator at the point of *P* may generate two kinds of motion. The motion is dynamic pull-in instability or jumping motion to the upper stable branch.

**Figure 8:**
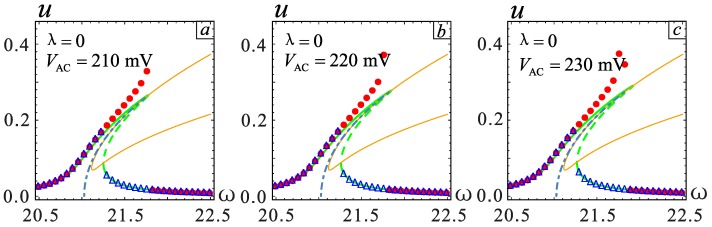
The changes in frequency response as AC voltage increases at *λ* = 0. (**a**) is the case of *V*_AC_ = 210 mV. (**b**) is the case of *V*_AC_ = 220 mV. (**c**) is the case of *V*_AC_ = 230 mV. The solid lines represent stable solutions. The dashed lines represent unstable solutions. The point represents the numerical solution.

**Figure 9:**
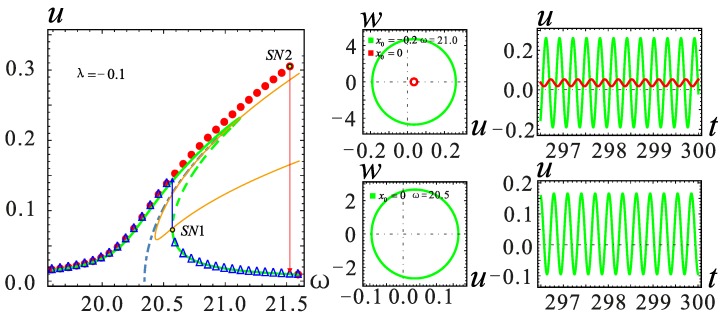
Phase diagram and the corresponding time history curve at *λ* = −0.1. The solid lines represent stable solutions. The dashed lines represent unstable solutions. The point represents the numerical solution.

The frequency response in the red frame is further considered for a detailed analysis; similarly, the two models for the *λ* = −0.1 and *λ* = 0.1 cases are selected for analysis. It can be seen from Figure 8 that in the case of section parameter *λ* = 0, when the AC voltage amplitude *V*_AC_ ≥ 0.22 V, the system shows a jump in the frequency response. The vibration amplitude becomes large with the increase of *V*_AC_, but the jump point does not change. The effect of the section parameter on jump phenomena is shown in Figure 9. The increase of *λ* will promote the occurrence of the jump phenomenon. On the contrary, when *λ* is small, a higher voltage is needed to observe a similar behavior. At the same time, the system will generate more energy output.

**Figure 10:**
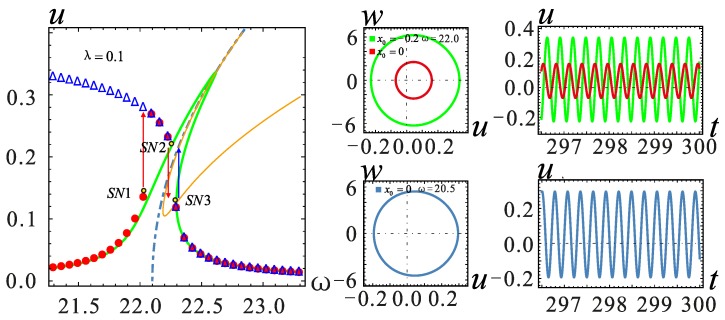
Phase diagram and the corresponding time history curve at *λ* = 0.1. The solid lines represent stable solutions. The dashed lines represent unstable solutions. The point represents the numerical solution.

**Figure 11:**
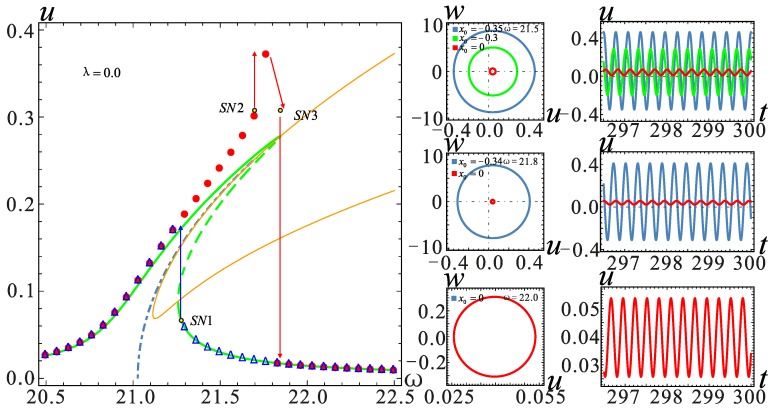
Phase diagram and the corresponding time history curve at *λ* = 0. The solid lines represent stable solutions. The dashed lines represent unstable solutions. The point represents the numerical solution.

The frequency is selected near the jump point in each case, and the corresponding time history curves are shown in Figure 9, Figure 10 and Figure 11. By setting different initial value *x*_0_, the displacement of all stable solutions can be obtained. If there is no hardening-to-softening behavior, the vibration of the resonator will appear from one stable solution to two stable solutions in the vicinity of the jump point *SN*1 as shown in Figure 9. The case of two stable solutions appears after jump point *SN*1. If the hardening-to-softening behavior appears, the solution case is the same as the one shown in Figure 9 when the nonlinearity is weak. On the contrary, in this situation, the two stable solutions case appears before the jump point *SN*1, as shown in Figure 10. When the nonlinearity is strong as depicted in Figure 11, there will be three stable solutions at most, and it changes into two stable solutions after the jump at the *SN*2 point. As the frequency increases, the stable solution finally returns to one.

## 5. Finite Element Verification

**Figure 12:**
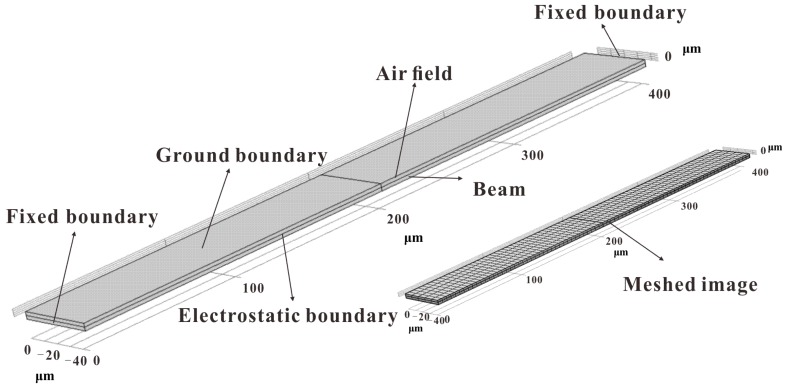
COMSOL simulation model.

Static and dynamic analyses of the system were carried out through the mathematical model in the previous research. The influence of surface machining error and gap distance on the nonlinear vibration was obtained. However, the results are obviously not convincing since the analysis was carried out using the mathematical model alone. In this section, the following physical quantities are assumed: *L* = 400 μm, *b* = 45 μm, *d* = 2 μm, *ρ* = 2.33 × 10^3^ kg/m^3^, *E* = 165 GPa, dielectric constant *ε*_0_ = 8.85 × 10^−12^ F/m and the clamped end thickness *h* = 2 μm. The finite element simulations of the several *λ* values are carried out using COMSOL software. The module used for this analysis is the MEMS module, and the electrical physical field interface is selected. The interface combines solid mechanics and electrostatics with the dynamic grids to model the deformation of an electrostatically-actuated structure. The number of degrees of freedom for solving this system is 31,185. Some nonlinear behaviors such as the pull-in effect and the electrostatic force softening effect are simulated in the real situation. The simulation of the pull-in voltage is carried out in the steady-state solver. The simulation of electrostatic force softening first proceeds through the parameterized scanning of DC voltage and then calculates the corresponding value of each point voltage in the steady-state solver and the eigenvalue solver. The physical model established through the finite element software is shown in Figure 12. As shown in the figure, below is the beam model and above is the air area.

**Figure 13:**
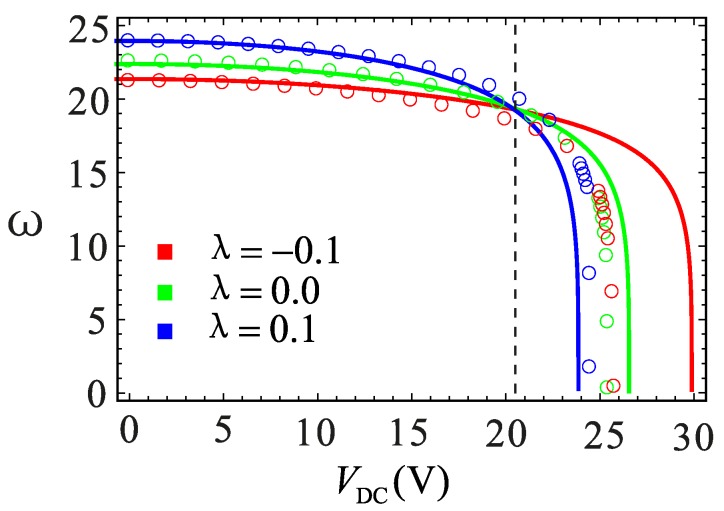
Relationship between DC voltage and equivalent frequency under different section parameters. The solid lines represent the analytic solutions. The circles represent the finite element solutions.

**Figure 14:**
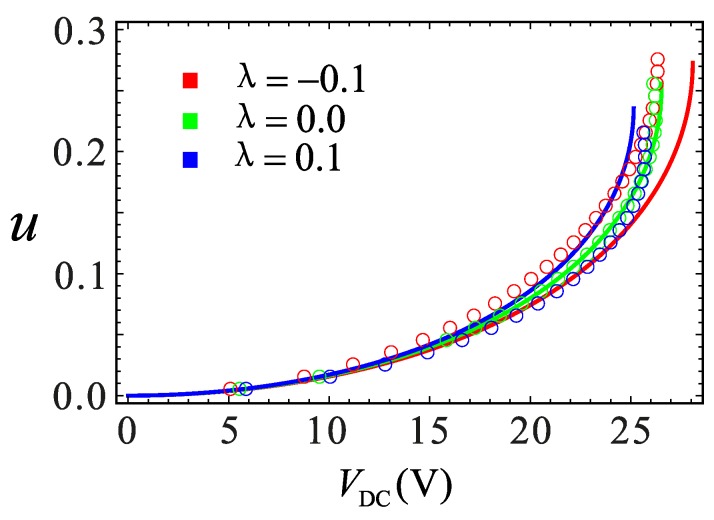
Relationship between DC voltage and static equilibrium point under different section parameters. The solid lines represent the analytic solutions. The circles represent the finite element solutions.

A comparison between the finite element simulation and analytical solution on the electrostatic softening effect is shown in Figure 13. Before *V*_DC_ = 20 V, the two results are in the good agreement. However, the error starts to increase near the pull-in position when the DC voltage exceeds 20 V. It is evident from these results that the error increases as the value of *λ* increases. The finite element simulation and analytical solution of the static pull-in effect are shown in Figure 14. It can be found that the pull-in position of the two results is same. The pull-in voltage is well simulated at *λ* = 0, while the other two cases are slightly different. It can be seen from Figure 13 and Figure 14 that the maximum error occurs near the pull-in point. The reason for the error could be as follows: the COMSOL software acquiescent structure stiffness is linear, while the actual system contains nonlinear stiffness. Although the analytical solutions take the nonlinear factors into account in the analysis, because of the limitation of MMS, an error between the analytical and numerical solution is inevitable especially when the amplitude is too large. The comprehensive mechanical behavior of the system cannot be obtained only through the numerical method. Therefore, the contradiction really needs further consideration, which is not within the scope of this paper. 

## 6. Conclusions

In this paper, the static and dynamic characteristics of a doubly-clamped electrostatic microresonator considering the effect of surface processing error on the thickness are studied. A section parameter is proposed to describe the microbeam changes in the upper and lower sections. The Galerkin discrete method is used to decouple, and the finite element analysis is carried out using the software COMSOL. To observe the system’s nonlinear vibration, the MMS is applied to obtain the approximate frequency response equation, and the long-time integral method is used to verify. The key conclusions are as follows.

(1) From derivation, the range of section parameter in micro resonators is *λ* ∈ [−0.3, 0.9].
(2) The occurrence of pull-in phenomenon could be promoted by *λ* increasing and *d* decreasing. Several typical cases are analyzed by using the potential energy curve and phase diagram. With either the increase of the parameter *λ* or the decrease of *d*, the barrier energy gradually decreases and the safe region reduces. As a result, the pull-in will occur.
(3) Under small perturbations, the resonator may vibrate in the neighborhood of the equilibrium point. When the gap distance is constant, the sectional parameter *λ* > 0 will make the system vibration tend to softening-type behavior. On the contrary, *λ* < 0 will make the system vibration lean towards hardening-type behavior. When the section parameter is constant, as the gap distance of the microbeam is larger, the hardening-type behavior more easily appears. Similarly, as the gap distance is smaller, the softening-type behavior is easier to obtain. Therefore, if the microresonator is thinner or thicker because of the surface machining error, the gap distance *d* can be adjusted to make the system vibration close to linear.
(4) The frequency response is obtained by MMS will lead to the nonlinear softening effect being weakened. This error is negligible when the amplitude of vibration is relatively small. As the amplitude increases beyond a certain value, this error will be more obvious. If the nonlinearity exhibits hardening-type behavior at the beginning, the nonlinearity of electrostatic force will gradually strengthen with the increases of the amplitude. Finally, the electrostatic force began to dominate when its nonlinearity effect on the system exceeded the influence of structural stiffness nonlinearity. At this time, the frequency response will exhibit hardening to softening behavior. The higher the value of *λ* is, the more easily it appears.

It can be concluded from the presented results that the surface processing error does affect the static and dynamic characteristics of the microresonator. When the existing micromachining process is not improved, it will go for a revision only after considering the processing errors in the original theoretically-based design. It can make the final product meet the theoretical design requirements and increase the rate of finished products.

## Figures and Tables

**Table 1:** Simulation cases under different parameters.

Case	*λ*	*d* (μm)	Dynamic Behavior
*P*_1_	−0.1	2	hardening-type vibration
*P*_0_	0	2	linear-like vibration
*P*_2_	0.1	2	softening-type vibration
*P*_3_	0	1.8	softening-type vibration
*P*_4_	0	2.0	hardening-type vibration

## Appendix A

(A1) $$g=\int_{0}^{1}A(x)\phi^{2}(x)dx$$

(A2) μ=c/g

(A3) $$k_{1}=\int_{0}^{1}{\left(I(x)\phi^{\prime \prime }(x)\right)}^{\prime \prime }\phi (x)dx/g$$

(A4) $$k_{3}=\int_{0}^{1}A(x){\left(\phi^{\prime }(x)\right)}^{2}dx\int_{0}^{1}\phi^{\prime \prime }(x)\phi (x)dx/g$$

(A5) $$\omega_{n}=\sqrt{k_{1}-3\alpha_{2}k_{3}{u_{s}}^{2}-\frac{1.8056\alpha_{1}}{{\left(1-1.48u_{s}-\delta \lambda \right)}^{3}}}$$

(A6) $$a_{q}=-3\alpha_{2}k_{3}u_{s}-\frac{4.00843\alpha_{1}}{{\left(1-1.48u_{s}-\delta \lambda \right)}^{4}}$$

(A7) $$a_{c}=-\alpha_{2}k_{3}-\frac{7.90997\alpha_{1}}{{\left(1-1.48u_{s}-\delta \lambda \right)}^{5}}$$

(A8) $$f=\frac{1.22\alpha_{1}\rho }{{\left(1-1.48u_{s}-\delta \lambda \right)}^{2}}$$
